# On the Uses and Influence of Mental Philosophy

**Published:** 1855-07-01

**Authors:** M. J. Rae

**Affiliations:** Physician to the Carlisle Dispensary.


### Original Communications.

### ON THE USES AND INFLUENCE OF MENTAL PHILOSOPHY.

BY M. J. RAE, M.D.

*Physician to the Carlisle Dispensary.*

THERE are few, if any, branches of study of such vital importance, that are so often wholly neglected, and so frequently little appreciated, as Mental Philosophy. Whilst our knowledge of the laws of nature has been continually progressing, and every year has been bringing fresh and valuable accessions to our previous acquaintance with the physical sciences,—mental science has, on the other hand, made comparatively little advancement. When we consider the marvellous progress which has been attained in the arts and kindred sciences, and that man has carried his researches so far into the arcana of nature, that she has been made to reveal herself in some of her most lovely forms and wonderful combinations; we are surprised that the science of mind—the science which treats of that curious instrument by which all these glorious results have been obtained—should have made little progress. The slow progression of mental science has been chiefly owing to that desire in the human mind, which is more particularly directed to the study of those branches that affect the physical wants—the comforts and convenience of mankind—rather than to those of a merely intellectual character. The prejudice against psychological investigations, and the mistaken notion of their utility, which exist in the minds of the majority of mankind, together with the generally acknowledged greater difficulties that are encountered in the study of mind than what are experienced in the study of matter, also partly account for the comparatively slow progress of mental science. Yet mental science has not been barren of cultivators. From the time of Thales, the Grecian sage, down to the present time, some of the greatest minds, and most profound thinkers that have ever lived, have been engaged in its elucidation. However, from many of these philosophers having started on wrong principles, and having attempted explanations of things beyond the province of the human intellect, their works have been productive of little or no benefit to mental science. Though many works on mental science will merely awaken wonder at their production and object in the minds of those who peruse them, and not a few will be buried in oblivion ; yet many writings on this science, both of ancient and modern times, distinguished for their great utility, and bearing upon them the stamp of true genius, will remain, through all time, the noblest productions of human reason, and imperishable monuments of departed greatness, of intellectual worth, of true nobility.

However much mental science may have been obscured, and its progress retarded by the subtilties, false theories, idle speculations, and mysticisms of authors, and by other causes just alluded to, it must nevertheless be admitted that the study of the human mind is one of the noblest and most important which can engage the attention of mankind. That on which Socrates discoursed, on which Plato reasoned, on which some of the ablest and best of men, in all ages, have exercised their thoughts, on which so much genius and philosophy, so much research, labour, and piety have been expended, cannot be indifferent to mankind in general. It surely concerns man to know the powers and susceptibilities of that Divine essence, that mysterious principle within him, which thinks, and wills, and reasons; which elevates him above the irrational creation, and gives him the mastery over the material world; which enables him to survey the past, and anticipate the future, and to rise from the contemplation of the finite to the infinite, from the visible to the invisible; which raises him to the semblance of an angel, or sinks him to the level of a fiend, and which is destined to endless woe, or to everlasting bliss. Shall man, then, carry his researches into every department of the arts, and attempt with unabated ardour to sink the "plummet of his intelligence" still deeper into the abyss of infinity, and watch the operations of nature in her varied and most secret haunts; and shall he not turn his thoughts, and observe the curious phenomena, the wondrous world of thought and action, that is constantly going on within himself,—phenomena, more curious, more wonderful by far than any which physical science or art can reveal, or that the busy artist, nature, throughout her wide and inexhaustible domain, can exhibit. It is no doubt necessary and useful for man to know the laws which govern the material universe, as well as to attend to the various arts which support and embellish life; yet "the proper study of mankind is man."

It is surely of the utmost importance to every man to be acquainted with the phenomena of mind, to be familiar with the laws which guide the operations of that instrument by which he acquires, retains, and applies knowledge. The laws of mind can be ascertained, as well as the laws of matter, not of course by the same kind of experiments, but what is just as deserving of that title, viz., by originating mental processes, either in our minds or in those of others; or they can be ascertained, as some writers say, by observation. A knowledge of the laws which govern matter or the material word is said to be power, but some distinguished writers have denied that knowledge is power in regard to an acquaintance with the laws of mind. But this is a narrow and altogether mistaken view of the matter. That knowledge of mind is power, history, biography, and the every-day occurrences of life alike determine. How many individuals that have made the past illustrious, whose names are "familiar in our mouths as household words," were mental philosophers? Almost every one of them. How many poor unknown sons of toil, by the elastic bound of genius, guided by a knowledge of mind, have raised themselves to fame and fortune, have swayed the empire of science, or directed the destinies of men? Let the history of the past witness! What is it that has given the Jesuits such unbounded influence over the fortunes of nations and the councils of kings? Is it their wealth, their numbers, or their force of arms? No, it is their knowledge of the human heart and mind. It is a mental and not a physical weapon which they wield—hence their power. Were they not trained to an intimate knowledge of the laws of mind, taught how to attack and storm the citadel of truth, how to direct the thoughts and mould the passions of men to their purposes, and were they not made masters of themselves, strong in purpose and resolve, by a training agreeable to the laws of mind, their mission to the world would be in vain, their influence over kings and states would crumble into nought; their institutions, their power, their very name would disappear from the earth!

What has enabled the present Napoleon to rise through the various vicissitudes of his life, through exile and the prison, through poverty, revolution, and the senate, to the imperial throne of France? What but his consummate tact, his intimate knowledge of the human mind, or at least with the French portion of it. What enabled the celebrated Abyssinian traveller, Bruce, to achieve the glorious discovery of the Nile's mystic source—a discovery, which had baffled the efforts of kings and conquerors, at the head of vast armies, to effect, and which the enterprise of men for three thousand years could not accomplish; but which he, a stranger, and alone, effected. Was it not the mighty and secret power which he wielded over the minds of the barbarians, making their ignorance, their passions, their prejudices, and their wills subservient to his purpose. His success will remain an almost unrivalled instance of the power of genius and personal intrepidity, guided by a knowledge of mind. It will remain a lasting triumph of the immeasurable superiority of moral over physical power —of mind over matter.

Instead, therefore, of knowledge of mind not being power, as some writers have asserted, it may safely be affirmed, that, if the phrase "knowledge is power" be applicable to any species of human knowledge, it is pre-eminently so to that which relates to an acquaintance with the laws of mind.

The extent and accuracy of an individual's information concerning external objects, his ability for acquiring a knowledge of his profession, and his capacity for properly directing the powers and faculties of his mind in any intellectual pursuit, will depend, in a great measure, on his knowledge of mind. Dr. Brown justly observes, that all science is in the mind. Science is just the comparison of phenomena, and the discovery of the order of their succession. It is the mind which compares, classifies, judges, reasons; and these comparisons, classifications, and reasonings, which are purely mental phenomena, constitute science. Without mind, science could not exist. The objects of science might exist without mind; the flowers might bloom, and the stars sparkle in the heavens, though there were no science of botany or astronomy to record their wonders.

It is impossible to make any satisfactory progress in the study of nature without some knowledge of mental philosophy. Although men may become well acquainted with the physical sciences, without paying much attention to the science of mind, yet they must always conduct their investigations according to the laws of mind, otherwise they will fail in arriving at satisfactory conclusions. The history of physical science proves this. Why was this branch of knowledge so barren of progress and improvement previous to the time of the illustrious Bacon? Because its cultivators—and they were both numerous, gifted, and energetic—did not pursue a method agreeable to the laws of mind. Bacon discovered a true mode of physical investigation. Hence the success which has marked the progress of the physical sciences since the discovery and application of the inductive method of philosophizing. A cultivated and well-ordered mind is of paramount importance to every man, but how shall he obtain such a desideratum without a knowledge of its laws? If a knowledge of the laws of nature be absolutely necessary before they can be safely and beneficially applied to practical purposes, it is surely as necessary that an individual should be acquainted with the laws which preside over his mental powers and moral emotions, before he can either rightly direct or properly cultivate them, so as to bring every faculty into the situation best calculated to favour its free and full expansion, and to guard against every circumstance that has a tendency to retard or prevent the complete and uniform development of the whole mind.

He who has paid little or no attention to the study of mind, and who has made no successful endeavours to analyse his intellectual powers and moral feelings, and is almost entirely ignorant of their springs and modes of action, is not very likely to have them in such a well-ordered condition as to favour his possessing a well-stored, a well-cultivated, and a well-regulated mind. Mental science ought to form an important part of the education of every man, that he may be better qualified to undertake the proper training of his intellectual faculties, and be more able to regulate and control the feelings, emotions, and desires of his mind; so that there may be a greater likelihood of his possessing the all-important advantage just alluded to, and of his attaining to the highest intellectual and moral elevation of which he is capable.

A knowledge of mind is necessary to the successful discharge of the duties and responsibilities of life. How shall parents, teachers, and guardians properly cultivate and discipline the minds and hearts of the children committed to their care, without a knowledge of mind? Without this, they may be crushing the opening mind, dwarfing the intellects of the youths, sowing the seeds of those vulture passions, or laying the foundations of intellectual and moral habits, which may ruin their happiness, and blast their prospects and usefulness in the world, and all the time be wholly unconscious of the dire mischief they are doing; nay, perhaps, be flattering themselves that they are discharging their important trust in the most praiseworthy and effective manner. Sad mistake! They who have best cultivated their own mental and moral powers, who have most closely analysed their own thoughts, emotions, and desires, and who have carefully studied the human mind, are the best qualified to undertake the charge and tuition of youth, to cultivate their intellectual and moral powers, to "pour the fresh instruction o'er the mind, and plant the generous purpose in the glowing breast." They are also best fitted to discharge other and higher duties of life.

Again, without a knowledge of mind, an individual is cut off from the enjoyment of many intellectual and refined pleasures. The higher order of poetry is placed beyond his reach. He merely knows, or attends to, the more common or every-day succession of his thoughts, and, consequently, can only appreciate the poetry which relates to these. He may therefore admire the simple song or the heart-stirring strains of the lyric, but he cannot appreciate or understand the sublime and lofty conceptions of Shakespeare. He may even consider the towering sublimity of Lear and the metaphysical subtilties of Hamlet overdrawn, unnatural, or meaningless. But they are as true to nature as the sentiment of the finest ballad. These characters have been drawn by the master-hand of genius, a genius which could fathom the depths and unravel the mysteries of the human heart and mind. The individual ignorant of the laws and constitution of the mind, is in the same predicament with regard to his ability to appreciate the truthfulness and power of these and other splendid creations of the great dramatist, as he would be of a painting, faithfully portraying a magnificent scene of classic Italy, had he never heard nor read of its sky, " so deeply, darkly, beautifully blue," nor of the splendour of its scenery, and knowing nature only as she appears in the cold and less genial north. Were a landscape, representing one of the finest Italian scenes by the glowing pencil of a Claude placed before him, he might probably admire its beautiful tints, its admirable light and shade, but he would conclude that it was overdrawn, not true to nature, the mere creation of the artist's exuberant fancy. It would be a correct representation of nature for all that; but depicting a part of nature which the individual did know, he could not therefore appreciate its beauty and correctness. So it is with the higher creations of Shakespeare and other great poets. The characters which they have drawn are true to nature, but they relate to regions and states of mind of which the individual is either ignorant, or unable to reach,—hence his inability to appreciate their beauty and truthfulness. To understand and value Shakespeare and kindred spirits, it is absolutely necessary to be thoroughly acquainted with the laws of mind, with the workings of the human heart, with the play of the passions, and with all the internal and complicated machinery of human thought and action.

The same holds good with regard to the higher order of painting. Without a knowledge of mind, an individual might admire and appreciate paintings, representing the ordinary thoughts and common occurrences of life, but the divine creations of the great masters would be to him little better than pieces of merely coloured canvas. The paintings of a great master, like the works of a great poet, may, however, affect a man without his understanding them, or knowing the cause of their power over him; it is because they are natural. He is affected by their sight, disturbed by " the joy of elevated thought," by the stirring of his deeper nature excited by the secret influence of the spirit of true genius. But were these masterpieces of the great artists not founded on nature, were they not constructed agreeably to the laws of mind as well as to the principles of art, the charm which they possess over the hearts of men would be dissolved; they would no longer be the shrines at which the poetic spirit of man delights to worship, nor possess the key to unlock the spirit of genius, nor kindle enthusiasm in the minds of our youthful artists, be to them incentives, guides, and finger-posts to fame and immortality. Knowledge of mind is not only necessary to understand the works of the great masters, but also to make any satisfactory progress in the higher walks of painting. The same remark applies, with all its force, to sculpture, oratory, and other arts.

Mental science greatly aids the cause of religion, by enabling the Christian to form clearer and more rational views of such difficult doctrines as free will, election, and other questions which divide the Christian world. Were mental science more cultivated, there would not be so many divisions among Christians with respect to these doctrines. Ignorance is the chief cause of the diversity of opinions held by men on many of the more difficult doctrines of religion. Many religious doctrines cannot be properly, if at all, understood by persons unaccustomed to psychological investigations. They may receive them as articles of a creed, but they cannot apprehend them by their understandings.

Mental science further aids the cause of religion, by enabling the believer to form a more rational and sublime conception of the personality and universality of the Deity. These are the highest and most overpowering subjects that can engage the attention of the human mind, and the most difficult to understand. Mental science, guided by the spirit of true piety, will enable the inquirer to approach them with a more rational hope of succeeding in forming an exalted idea of their nature and co-existence—an idea alike removed from pantheism on the one hand and a degraded personality on the other.

It has been asserted by some writers that mental science does not aid the religious spirit; but this is surely a mistaken idea. If piety, reverence, and love to God are deepened and exalted, as most assuredly they are by the study of His works, where, in all the wonders of creation is to be found such evidence of His wisdom and skill as is exhibited in the mind of man—the subject of the science? Were a spectator placed on some commanding elevation in the heavens, "far beyond the universe of stars," and able to survey the vast magnificence of the worlds and systems that circle in the infinity of space, amid all that glorious assemblage of material beauty and magnificence, and the innumerable host of worlds which would be revealed to his astonished gaze, he would find no object so wonderful and glorious as the human mind! When the contemplation of the stars, of the green fields, of the flowers, of the quiet lake and the ever-rolling ocean, deepens the religious feelings of man,—when the study of Nature raises man to the study of Nature's God, surely the study of the human mind, that which is superior to all nature, which is the masterpiece of creation, and an emanation from the Divine mind itself, is calculated to exalt the religious feelings, to give a deeper tinge to the piety, and a warmer glow to the love of the believer, and to raise him to a more sincere and humble adoration of the mighty majesty on high! Knowledge of mind, then, is the foundation of all improvement, the very basis of our intellectual and moral advancement; is an essential towards the possession of a well-cultivated and a well-regulated mind; is a chief cause of the great diversity of mental attainments observed among men of similar original intellectual endowments; and is the source of that tact, that practical knowledge of mankind, which is so indispensably necessary to success in the world. Knowledge of mind is also the means by which a man is raised still higher in the scale of intellect and morality, by which he is advanced to a larger participation in the higher and more exalted pleasures of life, and by which he is better fitted to discharge the duties and responsibilities of his station here, and prepared for a nobler and higher state of existence hereafter.

The preceding remarks chiefly refer to the advantages likely to arise to an individual through his knowledge of mind or mental science. We shall, however, conclude this part of the paper by taking a cursory view of the general influence of mental science—using this term in its widest sense—that is, comprehending both intellectual and moral philosophy; in short, all that relates to mind. First, look at the influence of mental science in every age! In ancient times, the philosopher was also the legislator, as Solon, Lycurgus, Thales, Socrates; and from the earliest periods to the present time, mental science or philosophy has influenced alike science, literature, legislation, and religion; nay, they have been grounded, so to speak, upon philosophy. The progress of each of them has been retarded or quickened, made beneficial or the reverse, according to the varying nature of the philosophy of the age. That this is no idle speculation will be seen by referring shortly to the history of the past. First, with regard to physical science. Allusion has been made already to the slow progress of this science during the many centuries that preceded the appearance of Lord Bacon. The peripatetic or Aristotelian philosophy, which held the sway in the schools during these ages, so cramped the energies and mind of man, that the efforts made by him to investigate nature failed, or produced worthless results. Bacon introduced the inductive method—a method more rational and better adapted to extend the sphere of human knowledge in regard to external nature. The deductive method has also been applied with great success to the investigation of natural phenomena.

Both these methods are the results of philosophy; they are founded on laws agreeable to the human mind. But philosophy not only influences the method of physical investigation, but also determines the object of research into the field of nature. The natural philosopher whose mind is imbued with a sensational philosophy, that is, a philosophy which declares that all mental states are the result of sensation, cultivates physical science with quite a different object in view from the philosopher who is influenced by an idealistic philosophy—that is, a philosophy based on a notion of self, with its native and exhaustless energies. To the former, nature is merely what it appears to the eye; he generalizes natural objects in their external relations only. The latter, also, accumulates facts relative to external objects, but he looks into nature to discover her hidden secrets—the forces which are everywhere in continual operation. "Nature," as it has been beautifully observed by Morrell, in his 'History of Modern Philosophy,' "is to the idealistic philosopher a glorious mystery, necessarily prompting us to the conception of spiritual agencies, which agencies are, in fact, only the 'indications of the Creator,' the varied forms in which a divine and spiritual power is diffusing itself throughout its own immense creation."

The influence of philosophy over physical science is still visible in the works of modern authors, and it will continue to exercise an influence over scientific research to the end of time. To those not familiar with the paramount importance of philosophy or mental science, we would recommend the perusal of Morrell's "History of Modern Philosophy;" the writings of Carlyle, Whewell, Humboldt, Herschel, &c. Look again at the influence of mental philosophy on literature. It is enough to allude merely to the historical writings of Hume, Gibbon, Macaulay, and Alison, to see the power which the philosophic tendencies of historical authors has over their respective productions. The poetry of Byron and Wordsworth affords a striking example of the effect of philosophy in moulding the thoughts and sentiments of poets, and leading them to an elevated or debased conception of man and nature. Byron has given some splendid descriptions of natural scenery—descriptions which, for power, beauty, and distinctness, have never been surpassed, if equalled, by any other poet, either ancient or modern. But graphic and minute as those poetical paintings are, they depict merely the outward and visible forms of nature. Wordsworth has also portrayed the outward forms of natural objects with great beauty and effect, but he has likewise endeavoured to discover in them the "good, the beautiful, and the true." To the former, nature appeared only a vast panorama of outward forms and beauty: to the latter, a world pregnant with living power and moral significancy. To the one, nature is only a magnificent assemblage of visible but isolated objects: to the other, a vast collection of varied forms, which link the spirit of man to the Spirit of the universe. Byron observed nature only as a poet: Wordsworth as a poet and a true philosopher. The latter has said, in his beautiful lines on Tintern Abbey,—

> "For I have learned to look on nature, not as in the
> Hour of thoughtless youth—but hearing oftentimes
> The still, sad music of humanity,
> Nor harsh, nor grating, though of ample power
> To chasten and subdue. And I have felt a presence
> That disturbs me with the joy of elevated thought,
> A sense sublime of something far more deeply interfused,
> Whose dwelling is the light of setting suns,
> And the round ocean, and the living air,
> And the blue sky, and in the mind of man,
> A motion and a spirit that impels
> All thinking things, all objects of all thoughts,
> And rolls through all things."

This is a key to his whole writings. Byron's popularity is already on the wane; and after ages will regret that a false and gross sensational philosophy and other causes, so perverted his mind and corrupted his heart as to prevent him leaving to posterity one piece really worthy of the greatness of his powers and the splendour of his genius. The poetry of Wordsworth is addressed to the deeper and purer feelings of man; and it will be read and admired so long as the human mind is affected by the beauties of nature, by purity of thought, and elevated sentiment. Wordsworth's mind, unlike Byron's, was imbued with an idealistic philosophy—hence the difference of their respective writings.

Let us here consider shortly the influence of mental philosophy on legislation and the condition of nations. To go no further back than the latter part of last century. We would ask, what chiefly gave rise to the horrible first French Revolution? It was most assuredly the promulgation of pernicious philosophical principles, until they spread through all ranks and classes of men, and corrupted the public mind of France. It was this, chiefly, which overthrew religion, uprooted virtue, and roused the fiendish passions that trampled on the throne, degraded liberty, outraged all order and decency, and made France red with the blood of her citizens. This tragic historical drama alone shows the influence of philosophy, and the absolute necessity that exists, at all times, for the cultivation and dissemination of a sound, healthy, and enlightened philosophy of mind among the people of every nation.

Whatever kind of philosophy exists in the minds of our Lockes, Adam Smiths, and Benthams, colours or determines their principles of social or political economy. The principles, political or philosophical, which are held by these philosophers, affect the public mind through books, the press, lectures, speeches. They affect the minds of the leading statesmen, who work them into a practical shape, and discuss and agitate them, until the public mind of a nation is affected by their truth or necessity, and legislative enactments are the result.

Philosophy also exerts a powerful sway over religion, not only over the religion of an individual, as in the case of Swedenborg, Priestley, and others, but over that of the Church. It has affected the religion of every Church in every age, modifying its opinions, its spirit, and its practices. The history of philosophy, as well as ecclesiastical and civil history, proves this. To take one example. It is the influence of a peculiar and somewhat mystical philosophy which is held by a few minds of the present day, that has produced the movement that is now going on in the Church of England. Whatever kind of philosophy is taught in the Universities, and imbibed by the theological and other students in attendance there, it will modify or give a colour to their religious opinions. Hence the necessity for a sound system of philosophy being taught in all Universities — without which, creeds and articles of religious belief will be of little avail in promoting sound and scriptural views of religion among those who are destined to become the teachers of others.

Seeing, then, the influence of philosophy over the opinions and actions of man, over science, legislation, literature, and religion, and seeing that in one form or another it has always held sway in the world, and that it will continue to do so till the end of time, exercising a useful or a baneful sway according to its nature, we must conclude that mental philosophy ought to form not only an important part of the education of man, but should, also, claim his attentive consideration during every period of his active existence. It ought to be his constant aim to attain to the knowledge of a sound, pure, and elevated philosophy of mind. Further, an individual may have some excuse for his being ignorant of many sciences, but can have none for his ignorance of mind. He may not be able to purchase a telescope to sweep the heavens, and aid his study of astronomy; and he may remain almost ignorant of the unseen world of life around him, from not being in possession of the microscope to reveal its wonders; and of chemistry, from his want of proper instruction and suitable apparatus. But he has the means within himself to become even a master in mental science. Unaided and alone, he may study mental phenomena as they are manifested within himself, or as they are exhibited in those around him. In addition to the careful study of the standard works on mental philosophy, the student should study mind practically as well as theoretically, and not confine his attention to books bearing expressly upon the subject. He ought to study the human mind as it is reflected on the page of history,—here, as in the world, he may obtain a practical knowledge of mankind, as well as become acquainted with the march of the human mind upwards through successive ages and civilizations to the present time, and also learn the various circumstances which influenced or retarded its progress and development. And he should study the mind as it is depicted by the powerful genius of Shakespeare, who has withdrawn the veil from the human heart, laid bare, with a master's hand, its hidden mysteries; explained the "subtilties of thought and the laws of passion"; who has presented us with a complete analysis of mind, with a perfect anatomy of the passions, emotions, and desires of man. And whether delineating the subtilties of love, the turbulence of passion, the rage of madness, or the chatterings of imbecility, or depicting any other phase of mind or character, he is equally natural, faithful, and profound. He is a metaphysician worthy of the closest study. The mind should be studied also as it is exhibited under the mild and genial sway of Christianity, and as it struggles for utterance through the gloomy atmosphere of infidelity, and as it is developed under the sway of the different religions and the different forms of political government. It should be studied among the rich and poor, the learned and unlearned, in its development, maturity, and decay; in health and in disease, at the festive board and at the hour of death. But, above all, the psychological student should study his own mind. It is here that his progress in mental science will be most satisfactory. For by attending closely to his own thoughts, emotions, and desires, and observing their springs and modes of action, by observing, in short, all the phenomena of mind as exhibited in himself, he may not only obtain a clear and comprehensive knowledge of the powers and susceptibilities of the mind, and of the laws which regulate their action, he may not only gain a knowledge of mental science, but, what is of more consequence, gain a knowledge of himself —a knowledge of his powers and capacities, his motives and desires, his virtues and defects,—a knowledge which will enable him to improve his intellectual powers, cultivate the affections of the heart, and give him the mastery of his will, make him firm in purpose and resolve, and fit him for the proper discharge of the duties and responsibilities of life.

